# Characterization of Glycerin Pitch and Its Application as a Plasticizer in Thermoplastic Starch Films

**DOI:** 10.1021/acsomega.5c12175

**Published:** 2026-08-08

**Authors:** Shin-Yong Yeoh, Shee-Shyuan Lam, Shi-Yu Khor, Kun-Yi Andrew Lin, Fei-Yee Yeoh

**Affiliations:** † Food Technology Division, School of Industrial Technology, 26689 Universiti Sains Malaysia, Persiaran Sains, Building G07, Gelugor, Pulau Pinang 11800, Malaysia; ‡ School of Materials and Mineral Resources Engineering, Universiti Sains Malaysia, Engineering Campus, Nibong Tebal, Pulau Pinang 14300, Malaysia; § Tekno Impact PLT, 1, Jalan Setia Sentral, Bukit Mertajam, Pulau Pinang 14000, Malaysia; ∥ Department of Environmental Engineering, 34916 National Chung Hsing University, 250 Kuo-Kuang Road, Taichung 402202, Taiwan

## Abstract

Glycerin pitch (GP), a low-value biodiesel byproduct, was characterized and evaluated as a potential plasticizer for thermoplastic starch (TPS) films in comparison with crude glycerin, laboratory-grade glycerol, and pharmaceutical-grade glycerol. GP was analyzed for its physical properties, elemental composition, and thermal behavior, and TPS films prepared with different glycerin grades were evaluated for visual appearance, thickness, mass, and morphology. GP exhibited strong alkalinity and a heterogeneous composition, including organic and inorganic constituents, with chloride-rich ash and elevated sodium and trace metals. Elemental analysis indicated that the organic fraction was predominantly glycerol with a relatively high oxygen content. TPS films plasticized with GP showed balanced plasticization, smooth surfaces, moderate elasticity, and the lowest solvent solubility among the samples. In contrast, films plasticized with pharmaceutical-grade glycerol exhibited overplasticization and structural defects at higher loadings. These results demonstrate that GP can function as an effective and low-cost plasticizer for TPS, improving film integrity and reducing solvent solubility while enabling the valorization of an industrial biodiesel byproduct. The findings highlight the potential of GP for use in starch-based biodegradable materials that require enhanced durability and controlled solubility.

## Graphical abstract

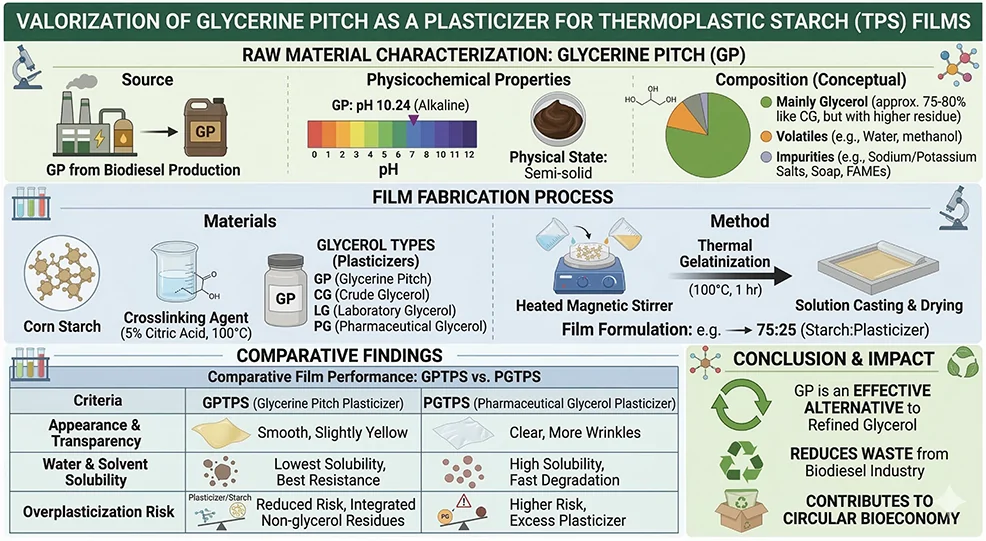

## 1 Introduction

The global surplus of crude glycerol has stimulated interest in its direct utilization and conversion into value-added products.^1^ Glycerin pitch (GP) is a scheduled waste byproduct generated during the purification of crude glycerol in biodiesel (fatty acid methyl ester) production from crude palm oil. It is typically disposed of via incineration or landfilling.^2,3^ GP generally contains 75–80% glycerol together with fatty acid residues, salts, water, and other organic impurities.^4^ It is produced at approximately 61.1 kg per tonne of biodiesel.^2^ With an estimated 101,000 tonnes generated annually, increasing biodiesel utilization is expected to elevate production to nearly 1.5 million tonnes per year.^3^ Incineration of GP not only releases toxic acrolein but also produces CO_2_ and aldehydes at a cost of ∼USD 800 t^–1^, while landfilling costs ∼USD 113 t^–1^ and risks soil and groundwater contamination.^5^ Consequently, the oleochemical industry is seeking sustainable valorization routes for GP to reduce waste management costs and support circular bioeconomy objectives.

Earlier studies have explored GP in various sectors, including liquid biofertilizers, polyhydroxyalkanoate (PHA) bioplastics, construction materials such as asphalt formulations, and carbon-based precursors and catalysts.^4,6–8^ However, its utilization as a plasticizer in polymeric systems remains largely unexplored. To date, the direct utilization of glycerin pitch from biodiesel refining as a plasticizer in thermoplastic starch systems has received very limited systematic investigation. Only limited studies have investigated GP as a substitute for refined glycerol in starch-based films, and the effects of such heterogeneous glycerin streams on film structure and performance remains insufficiently characterized.^9^ To date, a systematic evaluation of GP as a plasticizer in thermoplastic starch (TPS) films remains very limited. Furthermore, a comparative evaluation of glycerin pitch alongside multiple glycerin grades under identical processing conditions has not previously been reported. GP retains glycerol-like polyhydroxylated backbones and polar functional groups, suggesting potential as a low-cost plasticizing agent. Its heterogeneous composition, comprising salts, fatty acids, and organic residues, may also impart a lower hydrophilicity than refined glycerol, thereby improving moisture response and mechanical stability in polymer matrices.

TPS is a promising biodegradable polymer for use in packaging applications. It is inexpensive, abundant, and capable of forming continuous films with mechanical performance approaching that of petroleum-based plastics in suitable applications. Starch is stabilized by strong hydrogen bonding and requires high-boiling plasticizers such as glycerol, sorbitol, urea, or water to disrupt interchain interactions, lower the glass transition temperature, and improve flexibility.^10,11^ However, TPS remains hydrophilic and typically becomes brittle under dry conditions, while losing strength and barrier properties at high humidity. Plasticizer choice is therefore critical. For example, in PBAT/TPS blends, glycerol-plasticized TPS exhibits significantly lower strength and poorer interfacial cohesion than urea-plasticized TPS, in which cracks and voids are frequently observed under microscopy analysis.^12^ Moreover, pure glycerol is costly and highly hygroscopic, which limits long-term dimensional stability under humid storage.^13^ Cost-effective and sustainable alternatives to refined glycerol are, therefore, needed to improve biodegradable film systems.

Given its chemical similarity to glycerol and lower hydrophilicity, it is hypothesized that GP can function as an effective plasticizer for TPS, potentially improving flexibility, reducing water uptake, and enhancing structural integrity while lowering material costs. This study first characterized GP through physical, elemental, spectroscopic, and thermal analyses to evaluate its suitability as a plasticizing agent. TPS films incorporating GP were then fabricated and compared against films plasticized with crude glycerol (CG), laboratory-grade glycerol (LG), and pharmaceutical-grade glycerol (PG). The resulting films were analyzed for surface morphology, chemical interactions, solvent resistance, and water sensitivity to elucidate the plasticization behavior of GP within starch matrices. The overall aim is to establish the feasibility of GP as a functional plasticizer and to advance the valorization of industrial byproducts in the oleochemical and biopolymer sectors.

## 2 Materials and Methods

### 2.1 Materials

Glycerin pitch, crude glycerin, and purified glycerin were collected from IOI Acidchem Sdn. Bhd (Prai, Georgetown, Malaysia). Lab-grade glycerin (glycerol 85%) and citric acid were purchased from Merck (Rahway, United States). Nitric acid, starch, and sodium hydroxide were purchased from Sigma-Aldrich (St. Louis, United States). Ethanol (95%) was purchased from Chemiz (Shah Alam, Malaysia), and hydrochloric acid was purchased from Supelco (St. Louis, U.S.A.).

### 2.2 Characterization of GP

GP was visually inspected to document its color, texture, consistency, stickiness, softness, and physical state at room temperature. These observations were included as preliminary qualitative screening to describe the physical appearance and handling behavior of GP before detailed physicochemical characterization. Odor was also noted to identify any residual processing chemicals. The thermal behavior of GP was evaluated by heating 20 g of GP on a hot plate. This procedure was used as a preliminary evaluation of the thermal response of GP, rather than to determine precise melting or boiling points. Approximate thermal transition ranges were inferred from the temperatures at which liquefaction and bubbling were observed. Visual changes during heating and solidification upon cooling were recorded to assess reversibility. The density of GP was determined using a pycnometer. The pH of GP was measured using a DLAB digital pH meter (DLAB SCIENTIFIC, Senai, Malaysia) according to ASTM E70-19. A 10% w/v solution was prepared by dissolving 10 g of GP in deionized (DI) water and diluting it to 100 mL. Ash content was determined according to ISO 2096 using a CWF 1100 furnace (Carbolite Gero, London, United Kingdom), where 100 g of GP was heated in an alumina crucible at 750 °C for 3 h (at a rate of 10 °C/min). The measurement was repeated three times and reported as the mean value.

### 2.3 X-ray Fluorescence (XRF) Analysis of GP Ash

GP ash was ground into a fine powder using a mortar and pestle, then analyzed with an EDXRF Panalytical Minipal 4 (Malvern Panalytical, London, United Kingdom) to determine its compound composition.^14^

### 2.4 Carbon, Hydrogen, Nitrogen, and Sulfur (CHNS) Analysis

The CHNS content of GP was determined using a TruSpec Micro Analyzer (LECO Instruments, St. Joseph, United States) with a 1.98 mg sample. The oxygen content was calculated by subtracting the total C, H, N, S, and other elements from 100%.^1^

### 2.5 Inductively Coupled Plasma Optical Emission Spectroscopy (ICP-OES) Analysis

Trace elements in GP (Na, K, P, Ca, Mg, and Fe) were measured by using ICP-OES with an Optima 7300 DV spectrometer (PerkinElmer, Waltham, United States). A 1 mL melted GP sample was diluted to 10 mL with 1% HNO_3_. Because of sodium saturation, a 1000-fold dilution was applied. Results were compared with those reported in Armylisas et al.^1^

### 2.6 Fourier Transform Infrared (FTIR) Analysis of GP, CG, LG, and PG

FTIR analysis was performed using a Spectrum One FTIR Spectrometer (PerkinElmer, Waltham, United States) to identify functional groups and chemical characteristics of GP, CG, LG, and PG. Liquid samples were analyzed directly without further pretreatment. Spectra were recorded over the range of 4000–550 cm^–1^, with 32 scans at a resolution of 4 cm^–1^ under identical acquisition conditions for all samples. Background spectra were collected before each measurement to ensure baseline correction.^4^

### 2.7 Ultraviolet–Visible (UV–Vis) Analysis of GP, CG, LG, and PG

UV–vis spectroscopy was performed using a Cary 50 UV–vis spectrophotometer (Varian, Palo Alto, CA, United States). Each 1 mL sample was diluted to 10 mL with deionized water, and absorbance was measured across the range of 200 to 800 nm.

### 2.8 Thermogravimetric Analysis (TGA) of GP, CG, LG, and PG

TGA was performed using a Pyris 6 Thermogravimetric Analyzer (PerkinElmer, Waltham, United States) according to ref 1 with modifications. Approximately 10 mg of each sample was heated from room temperature to 800 °C at a rate of 10 °C/min under a nitrogen atmosphere.

### 2.9 Preparation of TPS Films

TPS films were prepared using 1.5 g of starch with defined amounts of plasticizer, citric acid solution, and deionized water as specified in Table 1. The casting solution consisted of 1 mL of 5% (w/v) citric acid, 10 mL of deionized water, and 0.5 g of glycerin-based plasticizer (LG, CG, PG, or GP). A control film without any plasticizer or glycerin was also prepared. Additional formulations containing 1 and 2 g of PG or GP were prepared to evaluate the effect of plasticizer concentration. The components were mixed and stirred at 300 rpm for 10 min, then heated in a 100 °C water bath with manual stirring until a viscous slurry formed. Approximately 10 g of the slurry was cast into a 10 × 10 cm silicone mold and dried at room temperature for 24 h to obtain TPS films. Sample designations and compositions are summarized in Table 1.

**Table 1:** Composition and Designation of TPS Films Prepared with Different Glycerin-Based Plasticizers^a^

Designation	Plasticizer type	Plasticizer mass (g)	Glycerin mass (g)	Starch (g)	DI water (mL)	5% Citric acid (mL)
Control	-	-	-	1.5	10	1
LGTPS	LG	0.5	0.5	1.5	10	1
CGTPS	CG	0.5	0.5	1.5	10	1
PGTPS	PG	0.5	0.5	1.5	10	1
PG1TPS	PG	0.5	1	1.5	10	1
PG2TPS	PG	0.5	2	1.5	10	1
GPTPS	GP	0.5	0.5	1.5	10	1
GP1TPS	GP	0.5	1	1.5	10	1
GP2TPS	GP	0.5	2	1.5	10	1

a “-” denotes absence of the ingredient in the formulation.

### 2.10 Characterization of TPS Films

The physical appearance and texture of the TPS films were visually examined before and after drying. Surface smoothness, color, and moisture were noted after casting. After 24 h of drying at room temperature, the films were demolded and assessed for wrinkles, elasticity, stickiness, and brittleness by sight and touch. Film thickness was measured at five points using a micrometer, and the average was calculated. Samples were conditioned in a dry room at room temperature, and care was taken to avoid any compression. Measurements were recorded in millimeters. The weight of each film was determined by using an electronic analytical balance after 24 h of conditioning at room temperature. Five film pieces from identical formulations were weighed, and the average was calculated. The measurement was repeated three times and is reported as the mean value.

### 2.11 Optical Microscope Analysis

Surface morphology was examined using an optical microscope with i-Solution software at 50× and 100× magnifications. Clean, unstained film sections on glass slides were analyzed for roughness, voids, starch dispersion, and phase compatibility.

### 2.12 FTIR Analysis of TPS Films

FTIR analysis was conducted using a Spectrum One FTIR Spectrometer (PerkinElmer, Waltham, USA) to identify functional groups and molecular interactions in the TPS films. Dried films were cut into flat rectangular specimens and analyzed directly without grinding or pelletization. The films had an average thickness of 0.192–0.361 mm, as measured with a digital micrometer (Section 2.11). Spectra were recorded in the range of 4000–550 cm^–1^ with 32 scans over approximately 2 min under identical acquisition conditions for all samples.

### 2.13 Solvent Solubility Analysis

TPS films were tested for solubility in DI water, 95% ethanol, 0.1 M HCl, and 0.1 M NaOH. Dried film samples (1 × 1 cm) were weighed and immersed in 5 mL of solvent at room temperature, then shaken at 200 rpm for 1, 6, 12, 24, and 48 h. After drying, solubility was calculated from weight loss. Only control, LGTPS, CGTPS, PGTPS, and GPTPS films were evaluated.

## 3 Results and Discussion

### 3.1 Physical Properties of GP

GP appeared as a dark brown, semisolid material with an oily texture and minimal odor (Figure 1). Previous studies have similarly described glycerin pitch as a dark brown material occurring in solid, paste, or gel-like forms.^1,2^ Its color and low transparency are attributed to the presence of water, inorganic salts, soaps, ash, and residual organic compounds.^15^ In addition, the elevated temperatures used during crude glycerol processing can promote thermal degradation of organic constituents and reactions such as caramelization and the Maillard reaction, further intensifying the material’s dark color.^16^

**Figure 1:**
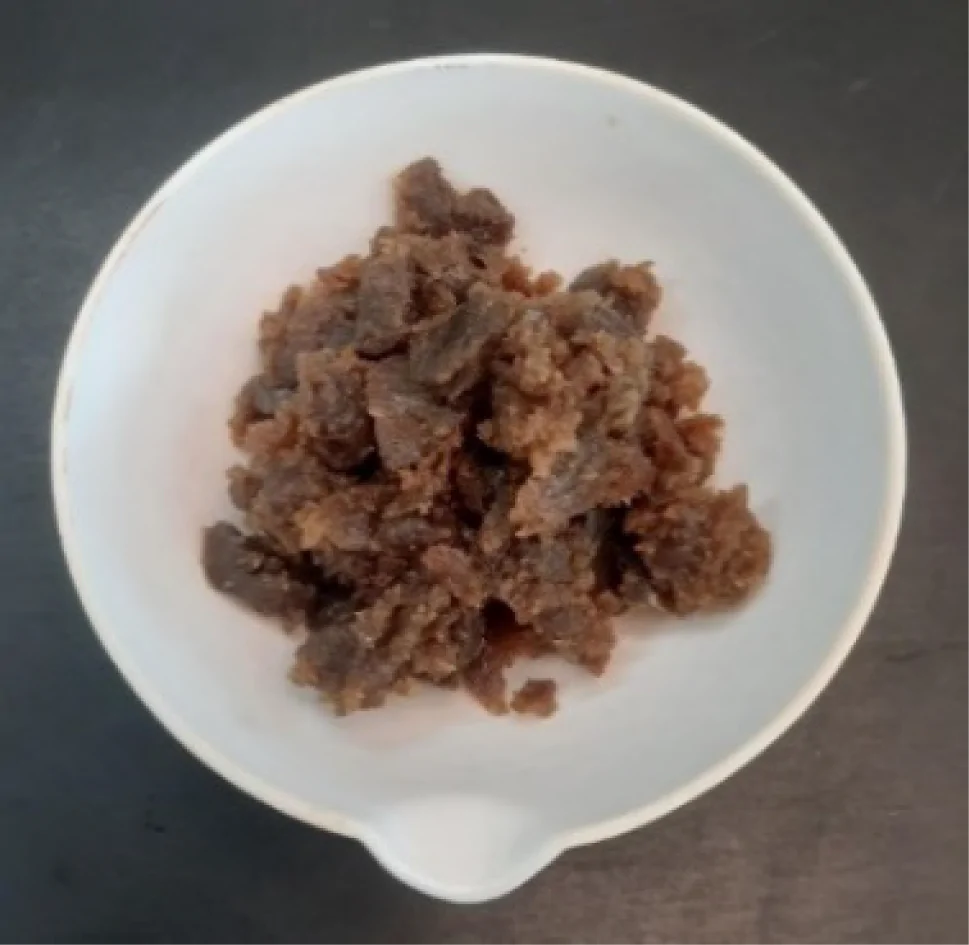
Physical appearance of GP.

GP softened and began to melt at approximately 80 °C, with complete liquefaction and vigorous bubbling observed above 250 °C, indicating the onset of boiling. Upon cooling to room temperature, the material resolidified into a semisolid state. This behavior is consistent with GP being a heterogeneous mixture containing glycerol, salts, soaps, fatty acid derivatives, and higher-molecular-weight residues. These components cause gradual softening at lower temperatures, followed by vigorous bubbling at higher temperatures. The bubbling likely arises from the release of moisture and volatile compounds, together with the thermal decomposition of organic constituents.^15^ Inorganic salts and soap phases may also act as nucleation sites that promote foaming and sustained bubbling. Progressive darkening and persistent bubbling are consistent with thermal degradation pathways, including Maillard reactions and caramelization at elevated temperatures.^16^ The resolidification of GP upon cooling indicates that part of the transition is physical in nature (thermoplastic flow), although irreversible chemical changes may occur at higher temperatures.

To contextualize these observations, the physicochemical properties of GP obtained in this study were compared with values reported in the literature (Table 2). The measured pH of 10.24 is consistent with previously reported values of 11.07–11.08 ^1^ and other studies reporting even higher alkalinity (11.83).^2^ This strong basicity is attributed to residual sodium-based catalysts, soaps, and salts originating from biodiesel or oleochemical refining processes. Variations in pH among studies likely reflect differences in feedstock composition, catalyst loading, washing or neutralization efficiency, water and ash content, and the degree of saponification during processing or storage. Compared with literature values for ash content (5.99–12.98%), the lower ash content measured in this study (4.53%) suggests reduced inorganic carryover, possibly resulting from improved washing or neutralization, cleaner feedstock, or more efficient phase separation. The greenish color of the ash suggests the presence of trace metal oxides or salts (e.g., Cu, Ni, or Cr), which may originate from catalysts or equipment wear. These species can catalyze oxidative or condensation reactions during heating, contributing to darkening and residue formation.

**Table 2:** Comparison of Physicochemical Properties of GP and Other Samples with Selected Literature

Physicochemical properties	GP	ref 1	ref 2	ref 17
Physical appearance	Dark brown semisolid paste	Dark brownish paste	Dark brown gel	Brownish solid
pH	10.24	11.8	11.83	>10
Ash content (%)	4.53	12.98	7.03	35

### 3.2 XRF Analysis of GP Ash

Table 3 presents the XRF elemental profile of the GP ash. The largest signals corresponded to aluminum oxide (Al_2_O_3_) and chlorine (Cl). Although Al_2_O_3_ appears as the most abundant species, this signal is primarily attributed to alumina crucible contamination during the ashing process rather than representing an intrinsic constituent of the GP ash. Accordingly, Al_2_O_3_ is reported for completeness but is not interpreted as a native inorganic component of the GP. After accounting for this artifact, chlorine emerges as the dominant inorganic component, consistent with the presence of inorganic chloride salts, predominantly sodium chloride (NaCl). These salts are typically formed during biodiesel refining when soaps are acidified, and residual alkalinity is neutralized. In conventional homogeneous base-catalyzed biodiesel production, this neutralization step generates salts that partition into the crude glycerol stream, explaining the chloride-rich ash observed in this study and highlighting the challenges associated with downstream purification. Moreover, homogeneous catalysis frequently produces soap and salt byproducts that reduce glycerol purity, whereas heterogeneous catalytic systems can reduce their formation.^17,18^

**Table 3:** Inorganic Elemental/Oxide Composition of GP Ash Determined by XRF (Excluding C, H, and Light Elements)

Formula (oxide or elemental form)	Concentration (%)
Al_2_O_3_	53.00
Cl	38.20
P_2_O_5_	3.94
SiO_2_	2.10
CaO	1.01
Fe_2_O_3_	0.60
NiO	0.48
K_2_O	0.28
PdO	0.19
Cr_2_O_3_	0.09
MoO_3_	0.07
CuO	0.06
MnO	0.03
Br	0.02
PbO	0.02
Au	0.01
Bi_2_O_3_	0.01
HgO	0.01
BaO	0.01
TiO_2_	0.01
Rb_2_O	0.00

The greenish color of the ash is likely attributable to transition-metal oxides detected by XRF, including NiO (0.48%), Cr_2_O_3_ (0.09%), and CuO (0.06%), which can impart a greenish-blue color even at subpercentage concentrations. Several trace-level species detected at concentrations close to the instrumental detection limit (e.g., Au, Bi_2_O_3_, HgO, BaO, TiO_2_, and other minor components) are reported for completeness only and should be interpreted with caution. These signals are near the detection threshold of the instrument and are not considered to be quantitatively significant. Overall, the XRF results characterize the inorganic composition of the ash fraction rather than the bulk GP material.

Radioactive or improbable elements below the detection confidence were excluded. Concentration values (%) are normalized to the detected inorganic ash components and do not represent bulk mass fractions of the original GP material. Light elements associated with organic matter (e.g., C, H, and O) are not quantified by XRF.

### 3.3 CHNS Analysis of GP

Table 4 presents the CHNS/O elemental composition of GP together with literature values reported in ref 1 for solid-paste and liquid glycerin. Carbon and hydrogen contents were 28.14% and 8.34%, respectively, while nitrogen and sulfur were below the detection limits. The oxygen content calculated by difference was 65.03%, indicating the presence of oxygen-containing species together with contributions from moisture and inorganic components not directly quantified by CHNS analysis.^19^ Compared with the theoretical composition of glycerol (C = 39.1%, H = 8.7%, and O = 52.2%), GP exhibits lower carbon and a higher apparent oxygen content. The elevated oxygen value is primarily attributed to the high moisture content of GP, consistent with the larger low-temperature mass loss observed in the TGA profile (Figure 4). Additional contributions arise from oxygen-containing inorganic species, including oxides and salts, detected in the ash and XRF analyses. Minor contributions from glycerol-derived oxygenates or polyglycerols may also be present, although these are likely secondary.

**Table 4:** Elemental Composition (C, H, N, S, O) of GP Compared with Ref 1 for Solid-Paste and Liquid Glycerin^a^

Elemental composition (%)	GP (this work)	Ref 1 (solid-paste GP)	Ref 1 (liquid GP)
C	28.14	34.8	23.91
H	8.34	7.76	5.74
N	BDL	0.2	0.25
S	BDL	0.01	0.01
O	65.03	57.23	70.09

a BDL, below the detection limit.

Compared with the values reported in ref 1, the carbon content of GP (28.14%) lies between the reported solid-paste (34.8%) and liquid (23.91%) forms, while the hydrogen content (8.34%) exceeds both literature values (7.76% and 5.74%), indicating a composition more closely associated with glycerol-rich material. The oxygen content (65.03%) similarly falls between the literature values for the solid-paste (57.23%) and liquid (70.09%) fractions. Notably, nitrogen and sulfur were not detected in the present sample, whereas ref 1 reported low but measurable levels (N = 0.20–0.25%; S = 0.01%). This suggests that the GP analyzed in this study contains fewer nitrogen- and sulfur-bearing impurities. Overall, the CHNS profile indicates a glycerol-dominated organic matrix together with additional oxygenated constituents and minor inorganic species not detectable by CHNS, consistent with the ash and XRF analyses.

### 3.4 ICP-OES Analysis

ICP-OES analysis identified Na, K, P, Ca, Mg, and Fe in the GP sample (Table 5). Sodium was the most abundant element, measured at 1.861 ppm, which exceeds the values reported in ref 1 for both the solid-paste (0.857 ppm) and liquid (0.465 ppm) fractions. The elevated sodium concentration is consistent with inorganic carryover from biodiesel refining processes, particularly the use of alkaline catalysts, such as NaOH or NaOCH_3_. During processing, saponification and subsequent acidification or neutralization steps generate sodium salts and carboxylates that can partition into the crude glycerol stream, contributing to the inorganic fraction of glycerin pitch. Other metals were detected at much lower levels, including K (0.010 ppm), P (0.012 ppm), and Ca (0.026 ppm). Magnesium (0.004 ppm) and iron (0.003 ppm) were also detected in the present sample but were reported as below the detection limit in ref 1. This indicates slightly higher trace-metal content in the GP analyzed in this study. Although these concentrations are minor relative to the bulk composition, divalent cations such as Ca^2+^ and Mg^2+^ may form sparingly soluble soaps that can contribute to deposits during filtration or thermal processing.

**Table 5:** Comparison of GP Metal Elements Measured by ICP-OES (ppm) with Ref. 1 for Solid-Paste and Liquid GP^a^

Metal element (ppm)	GP (this work)	Ref 1 (solid-paste GP)	Ref 1 (liquid GP)
Na	1.861	0.857	0.465
K	0.01	0.007	0.006
P	0.012	0.005	0.018
Ca	0.026	0.023	0.02
Mg	0.004	BDL	BDL
Fe	0.003	BDL	BDL

a BDL, below the detection limit.

Overall, the ICP-OES results complement the CHNS analysis by revealing the inorganic fraction of the GP. While the organic elemental composition is broadly consistent with literature values, the present sample exhibits a higher sodium content and detectable Mg and Fe, likely reflecting differences in feedstock composition and refining or washing efficiency.

### 3.5 FTIR Analysis of GP, CG, LG, and PG

Figure 2 presents the FTIR spectra of GP, CG, LG, and PG. All samples exhibit the characteristic absorption profile of glycerol-based systems.^1^ A broad and intense O–H stretching band centered around 3275 cm^–1^ indicates extensive intermolecular hydrogen bonding typical of polyhydric alcohols. Such broadening and a shift toward lower wavenumbers within the 3580–3200 cm^–1^ region are consistent with hydrogen-bonded hydroxyl groups.^20^ Aliphatic C–H stretching vibrations are observed at 2935 cm^–1^ (asymmetric CH_2_) and 2881 cm^–1^ (symmetric CH_2_), while CH_2_ bending appears at 1417 cm^–1^. Strong C–O stretching bands between 1100 and 1000 cm^–1^ correspond to primary and secondary alcohol functionalities within the glycerol backbone. These bands fall within the typical spectral range reported for alcohol groups and confirm the presence of glycerol-derived structures in all samples. Additional bands at 994 cm^–1^ and 850 cm^–1^ are attributed to C–C skeletal vibrations and C–C–O symmetric stretching associated with glycerol molecules.^1,20^

**Figure 2:**
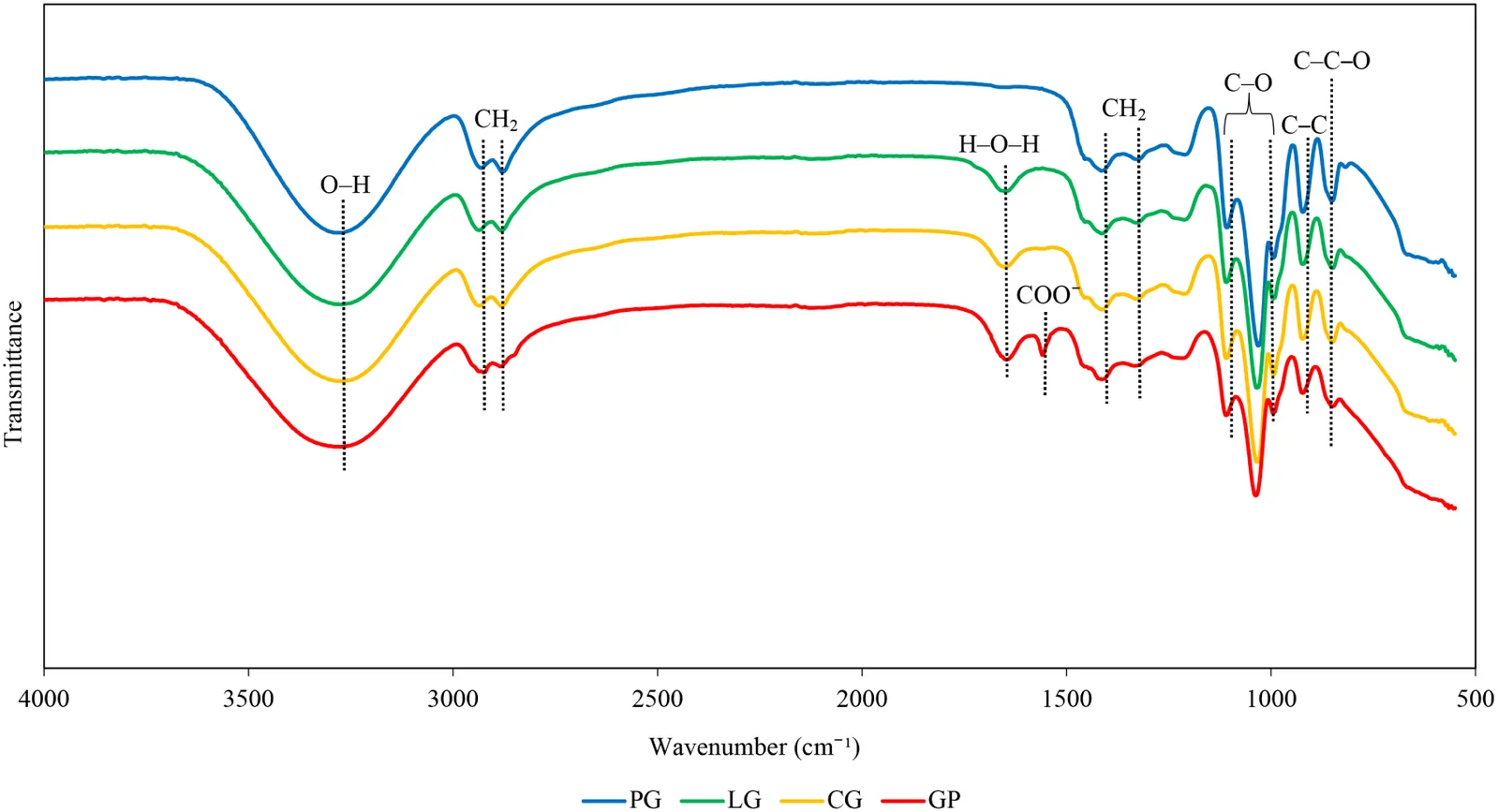
FTIR spectra of glycerin samples (GP, CG, LG, and PG).

In addition to the characteristic glycerol bands, GP exhibits a distinct absorption at 1558 cm^–1^, which is absent in CG, LG, and PG. This band lies within the asymmetric stretching region of carboxylate ions (COO^–^) (1550–1610 cm^–1^) and is consistent with the presence of carboxylate salts derived from soap residues formed during saponification and neutralization in biodiesel refining^1,20,21^ A band at 1649 cm^–1^, attributed to the H_2_O bending vibration, is observed in CG, LG, and GP but is absent in PG. The stronger intensity of this band in the less-purified samples suggests a higher water content.^1,20^ Overall, the FTIR spectra indicate that GP is predominantly glycerol-based, with measurable water and trace amounts of carboxylate-derived impurities.

### 3.6 UV–Vis Analysis of GP, CG, LG, and PG

Figure S1 shows the color appearance of the diluted glycerin solutions, while Figure 3 presents the corresponding UV–Vis spectra. GP exhibits the most intense yellow color and the highest absorbance across the measured wavelength range, with a pronounced increase in absorbance below 500 nm, extending toward 300 nm. CG follows a similar spectral trend but with approximately half the intensity of GP. In contrast, LG and PG are colorless and remain near the baseline across most of the spectrum. PG shows negligible absorbance between 320 and 800 nm, while LG remains near the baseline from 380 to 800 nm, with only a slight increase in the near-UV region.

**Figure 3:**
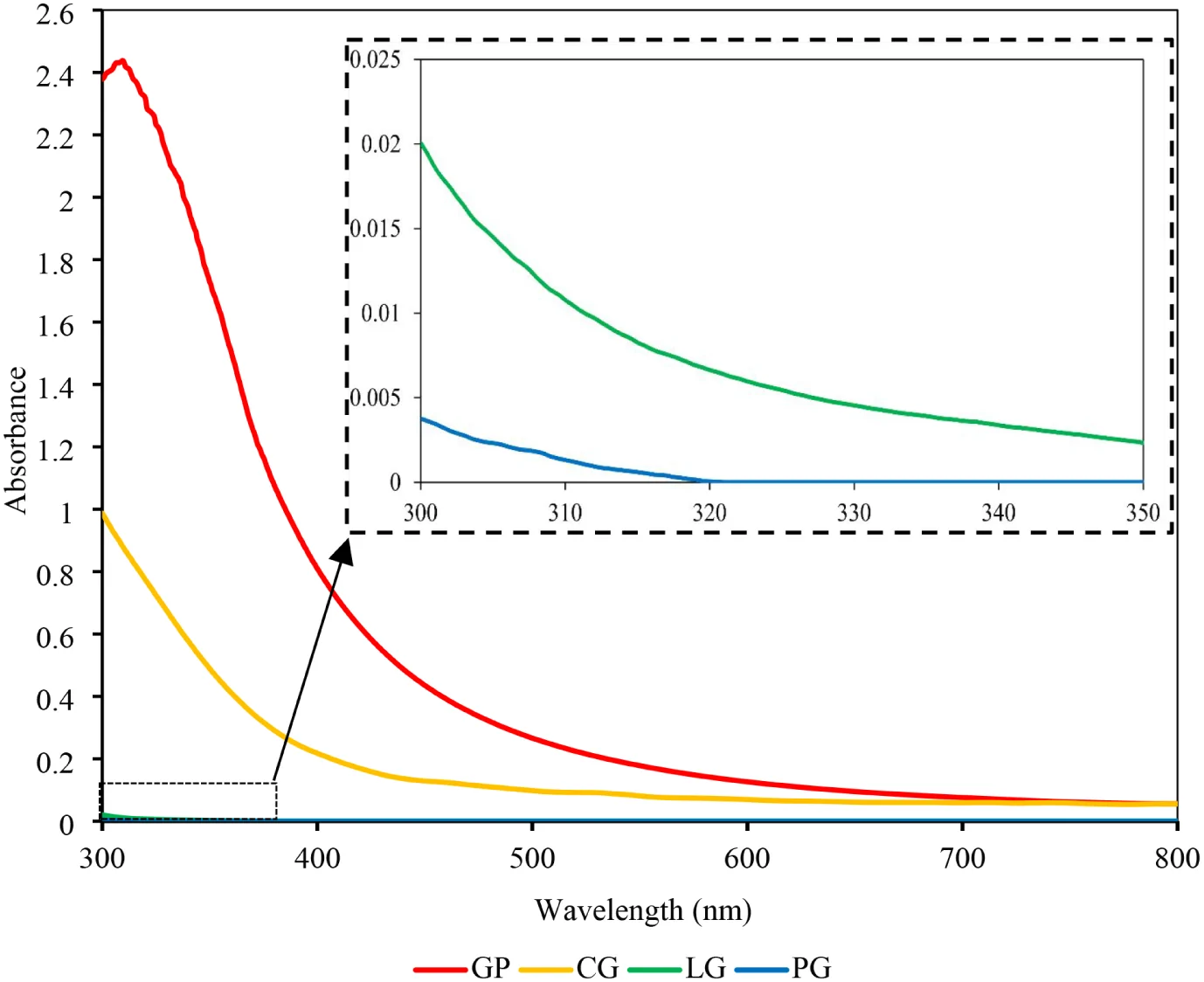
UV–vis spectrum of glycerin samples (GP, CG, LG, and PG).

These spectral patterns indicate that GP and, to a lesser extent, CG contain chromophoric species that absorb in the UV–visible region. Such absorption is consistent with degradation products derived from pigments originally present in crude palm oil, including carotenoids and chlorophyll derivatives. During high-temperature refining, these pigments can undergo thermal and oxidative degradation to form compounds such as apocarotenoids and pheophytin-type derivatives, which possess extended conjugated systems and absorb strongly in the UV–visible region, contributing to the darker color of GP.^22,23^ The moderate absorbance observed for CG is consistent with the presence of residual impurities commonly found in crude glycerin, including oxidized byproducts, soaps, ash, and trace pigments. In contrast, LG and PG exhibit minimal absorbance, confirming their higher purity. The slight increase observed in the deep-UV region is likely associated with intrinsic electronic transitions of glycerol, particularly O–H- and C–O-related transitions.

### 3.7 TGA of GP, CG, LG, and PG

Figure 4 presents the TGA profiles of GP, CG, LG, and PG. Both GP and CG exhibited three distinct degradation stages, whereas LG and PG showed simpler two-stage and single-stage behaviors, respectively. In Stage I (25–105 °C for GP; 25–140 °C for CG), a pronounced mass loss is observed due to the volatilization of water, methanol, and other low-boiling components. This behavior is consistent with reported assignments for glycerin pitch derived from palm-based biodiesel residues^1^ and with TGA profiles of glycerol systems that display a minor initial mass loss associated with residual moisture and trace volatiles before the main glycerol degradation event.^24^ GP exhibits a greater Stage I mass loss (23.72%) than CG (12.81%), indicating a higher volatile fraction and incomplete purification. This interpretation is consistent with reports that crude glycerol from biodiesel transesterification frequently contains substantial MONG (matter organic nonglycerol) components, including methanol, water, soaps and salts, free fatty acids (FFAs), fatty acid methyl esters (FAMEs), residual glycerides, and other organic species.^25^ TGA studies of crude glycerol and related biodiesel-derived fractions typically show an initial volatilization stage dominated by methanol and moisture evaporation, followed by a main decomposition stage associated with glycerol and FAME/glyceride fractions.^24^ The presence of these volatile impurities explains the pronounced multistage degradation behavior observed for GP and, to a lesser extent, CG, whereas the more purified LG and PG exhibit a simpler weight-loss profile characteristic of purified glycerol.

**Figure 4:**
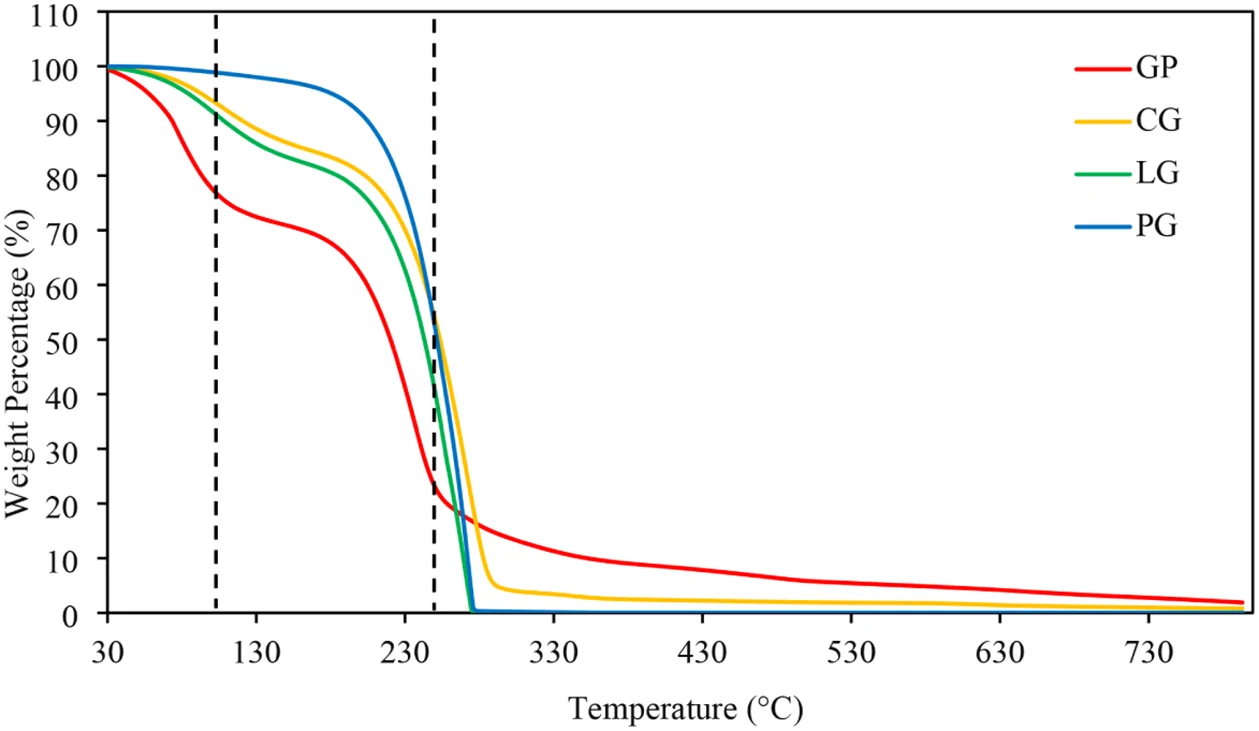
TGA of GP, CG, LG, and PG.

The second stage (105–260 °C for GP; 140–295 °C for CG) corresponds to the primary thermal decomposition of glycerol, resulting in 56.73% and 82.70% mass loss, respectively. This confirms glycerol as the dominant constituent in both samples, although the lower value observed for GP reflects dilution by impurities and heavier residues. This stage is consistent with the thermal behavior of pure glycerol, which typically exhibits a single dominant decomposition event with a T_max_ of approximately 211–238 °C.^24^

In Stage III (260–800 °C for GP; 295–800 °C for CG), the slower mass loss is attributed to the degradation of heavier and less volatile constituents, including FAMEs, glycerides, FFAs, and soap-derived salts. The higher-temperature degradation ranges reported for FAMEs (T_max_ = 312–336 °C) and rapeseed oil (277–483 °C) support this assignment.^24^ GP leaves the highest residual mass (1.88%) compared with CG (0.76%), which is attributed to inorganic ash and thermally stable polymerized residues. Incomplete volatilization and overlapping decomposition events leading to stable byproducts have been reported for glycerol systems containing alkaline species (e.g., NaOH or alkoxides),^26^ which is consistent with the impurity-rich composition of glycerin pitch derived from palm-based biodiesel streams.^1^

In contrast, LG and PG exhibit much simpler degradation behavior. LG shows two distinct stages: an initial mass loss attributed to moisture evaporation (14.84%), followed by glycerol decomposition (85.16%), with no detectable residue, consistent with its approximate composition of 85% glycerol and 15% water. PG shows a single sharp mass-loss event between 25 and 280 °C, corresponding to complete volatilization, confirming its high purity. Overall, the TGA profiles clearly distinguish the samples by their purity and thermal complexity: PG and LG show relatively clean and homogeneous thermal behavior, CG contains moderate impurities, whereas GP exhibits the most complex decomposition pattern and the highest residual mass due to inorganic and polymerized constituents.

### 3.8 Characteristics of TPS Films

Figure 5 shows the cast TPS slurries immediately after manual spreading into the molds, before drying. Across the formulations, the surfaces exhibited similar textures with visible moisture. Samples containing higher plasticizer loadings (PG2TPS and GP2TPS) appeared wetter and smoother than those with lower plasticizer content, consistent with the known effect of increased glycerol fractions on slurry flow behavior and plasticization of starch systems.^27^ GPTPS, GP1TPS, and GP2TPS displayed a light-yellow coloration that intensified with increasing GP content. This observation is consistent with the intrinsic color of palm-derived crude glycerol and glycerin pitch, which contain residual pigments and impurities originating from biodiesel processing streams.^28^ None of the cast films exhibited a completely smooth or flat surface. This irregularity is attributed to manual spreading using a glass slide together with the relatively high viscosity of the starch slurry before drying.

**Figure 5:**
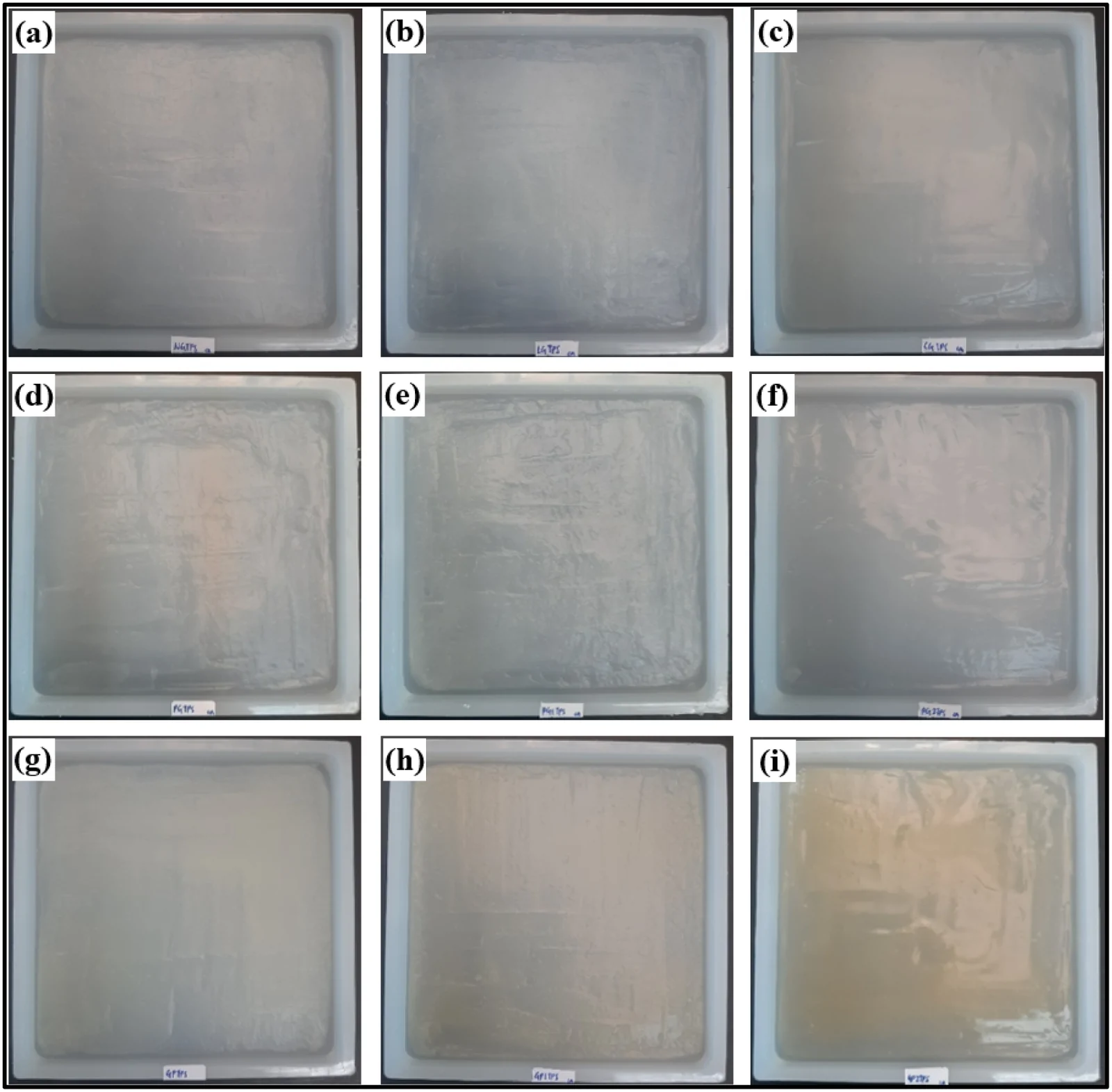
Casted TPS samples before drying. (a) Control, (b) LGTPS, (c) CGTPS, (d) PGTPS, (e) PG1TPS, (f) PG2TPS, (g) GPTPS, (h) GP1TPS, and (i) GP2TPS.

Figure 6 presents the dried and demolded TPS films after 24 h of drying. Among the formulations, GPTPS exhibits the most uniform appearance, characterized by a relatively smooth surface with minimal wrinkling, moderate elasticity, and a nonsticky texture. This behavior suggests that GP provides balanced plasticization and drying behavior, likely due to its viscosity and heterogeneous composition, without excessive moisture retention. PGTPS shows comparable film formation but exhibits slightly greater surface wrinkling, although the overall drying behavior, elasticity, and tack remain acceptable. In contrast, the control film is dry and highly brittle, exhibiting pronounced wrinkling and dimensional distortion during drying, consistent with poor film formation in the absence of a plasticizer.^29^ PG2TPS (highest PG loading) appears the wettest after drying and exhibits the greatest elasticity and surface tack, indicating overplasticization and reduced drying efficiency. Overall, increasing the glycerin plasticizer content enhances moisture retention and elasticity but may also increase surface tack. Among the plasticizers tested, GP produced films with the most favorable balance of surface smoothness, moderate elasticity, and low tack, indicating improved film integrity.

**Figure 6:**
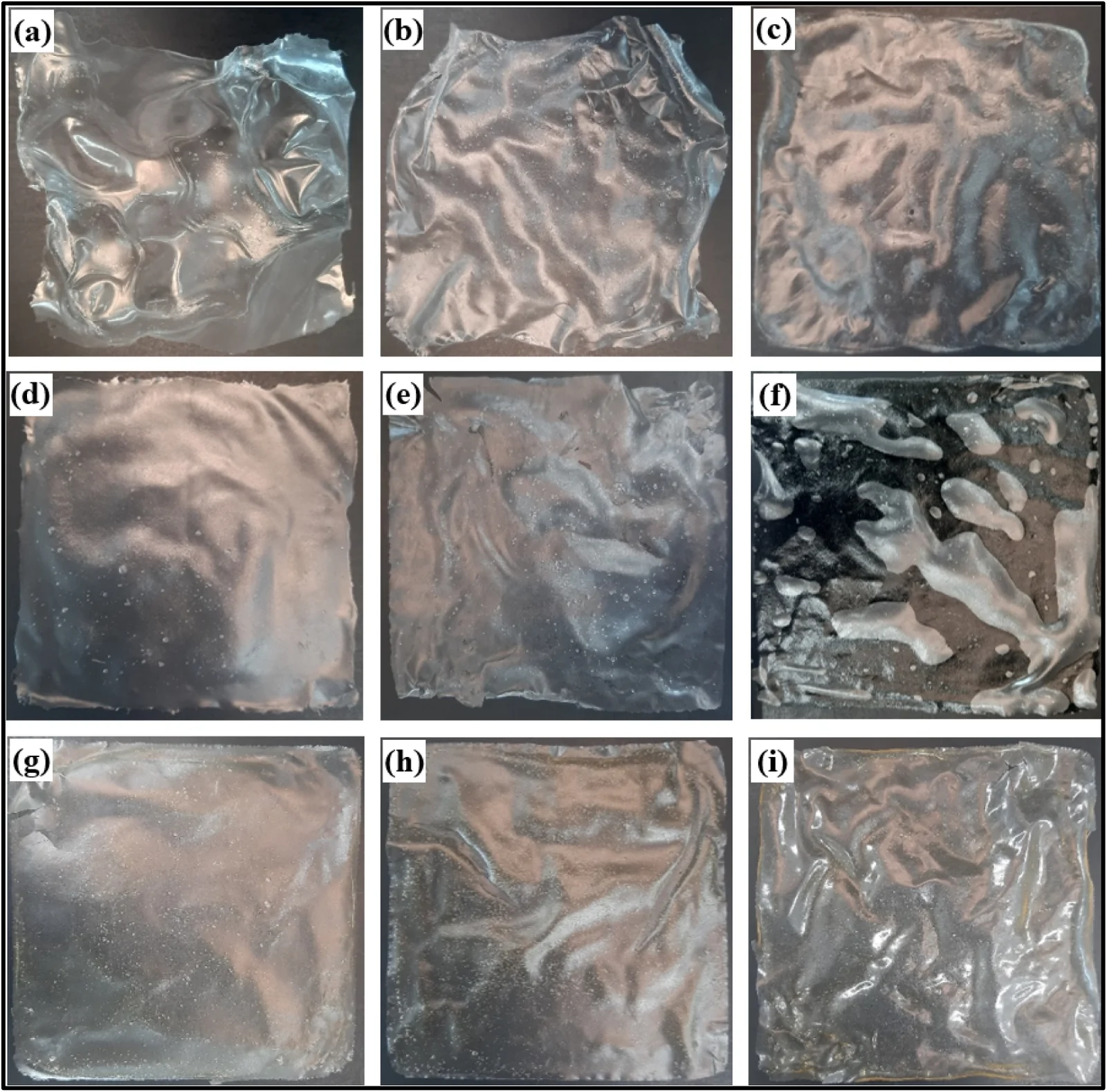
Dried, demolded TPS films: (a) control, (b) LGTPS, (c) CGTPS, (d) PGTPS, (e) PG1TPS, (f) PG2TPS, (g) GPTPS, (h) GP1TPS, (i) GP2TPS.

Because all films were prepared using a fixed casting volume and mold area (approximately 10 mL of solution cast into a 10 × 10 cm mold), the initial wet-layer thickness was estimated to be about 1 mm. During drying, substantial thickness reduction occurred due to water evaporation and shrinkage of the polymer matrix. Consequently, the final dry film thickness was governed primarily by the nonvolatile fraction of the formulation (starch, plasticizer, and other nonvolatile constituents) together with the extent of shrinkage during drying. Figure 7a presents the thickness of the different TPS films. Film thickness is an important parameter influencing mechanical performance and water vapor permeability,^30^ and it is often used as an indicator of barrier properties, optical clarity, and structural integrity in starch-based films.^31^ Under the fixed casting conditions employed in this study, variations in final thickness therefore reflect differences in formulation composition rather than differences in casting geometry.

**Figure 7:**
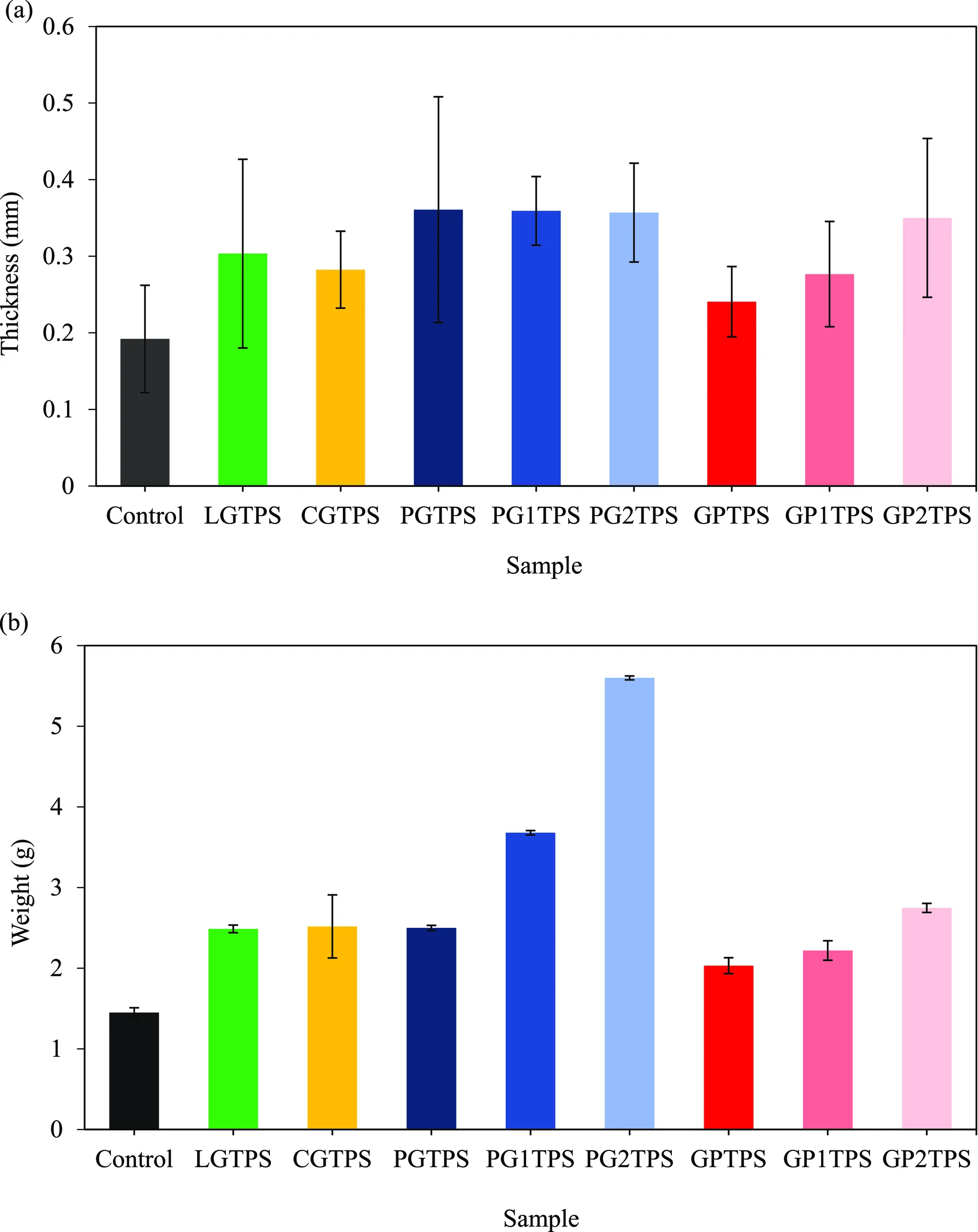
(a) Thickness and (b) weight of various TPS films.

Among the formulations, PGTPS exhibited the greatest thickness, whereas the control film was the thinnest (Figure 7A). However, these differences were modest and should be interpreted as secondary effects rather than intentional controls of film thickness. Because both the casting volume and mold area were fixed, the overall film thickness was primarily constrained by the casting protocol. The small variations observed are therefore attributed to differences in solids content, moisture retention, slurry viscosity, and drying shrinkage under otherwise identical casting conditions, rather than to changes in film geometry. Higher glycerol levels likely increased slurry viscosity and water retention, which influenced drying behavior and resulted in minor differences in the final dry thickness.^30,31^ Similarly, the slightly greater thickness observed for GP-plasticized films at higher GP loadings may arise from the presence of impurities (e.g., MONG, fatty acids, and ash), which alter slurry viscosity and effective solids content, thereby affecting drying shrinkage rather than the initial casting thickness.

Figure 7b presents the masses of the TPS films. PG2TPS exhibited the highest mass, whereas the control film showed the lowest. Across the plasticized formulations, the mass ranking was CGTPS > PGTPS > LGTPS > GPTPS > control. Films plasticized with glycerol typically exhibit higher mass because the plasticizer contributes additional solids and, due to its strong hygroscopicity, promotes moisture retention within the polymer network, thereby increasing equilibrium water content. Previous studies on starch-based films similarly report greater moisture uptake and associated property changes with increasing glycerol content.^31^ Increasing the plasticizer loading further increased the film mass in both the PG and GP systems. This effect was more pronounced for PG, consistent with the more efficient incorporation and water-binding capacity expected for higher-purity glycerol.^32^ In contrast, GP-plasticized films tended to exhibit lower mass than their PG counterparts at comparable loadings. This behavior is consistent with the heterogeneous composition of glycerin pitch, which commonly contains impurities such as water, soaps, free fatty acids, ash, MONG (matter organic nonglycerol), and occasionally residual methanol. Some of these components can volatilize or degrade during drying, reducing the amount of retained material in the final film.^1^

### 3.9 Optical Microscope Analysis of TPS Films

At 50× magnification (Figure 8), GPTPS and LGTPS exhibit slightly lower wrinkle density, whereas PG2TPS displays the most pronounced surface undulations. Increasing plasticizer loading generally amplifies surface waviness, most clearly in PG-plasticized films and, to a lesser extent, in GP-plasticized films. In contrast, the control and PGTPS films exhibit comparatively shallow wrinkling. Moderate glycerol incorporation is known to improve matrix consolidation and surface smoothness in TPS films, whereas excessive plasticizer levels can induce softening and plasticizer migration, leading to increased waviness and surface defects.^33^ At 100× magnification (Figure 9), PGTPS exhibits the most uniform and consolidated matrix, characterized by relatively few dark inclusions and sparse bright particulates. The dark features likely correspond to voids or entrapped air pockets, while the bright particulates may represent partially ungelatinized starch granules. This observation is consistent with reports that moderate glycerol levels improve matrix continuity and reduce surface roughness in starch films and TPS-based blends.^34^ In contrast, PG2TPS shows a folded and rough surface, while LGTPS exhibits the highest density of dark inclusions and bright particulates, followed by CGTPS. The observed microstructural features are strongly influenced by both plasticizer identity and concentration. Moderate glycerol levels generally enhance film morphology by improving starch–plasticizer interactions, whereas excessive plasticizer loading promotes oversoftening, plasticizer migration, and defect formation.^35,36^ Higher glycerol levels can also increase solution fluidity during casting, potentially facilitating air entrapment and leading to bubbles or pores after drying. Conversely, moderate plasticizer levels tend to produce more uniform and consolidated film structures.^37^ These observations are consistent with previous studies showing that glycerol-plasticized TPS often develops cracks and voids at higher plasticizer loadings, reflecting reduced matrix cohesion and incomplete plasticization.^12^

**Figure 8:**
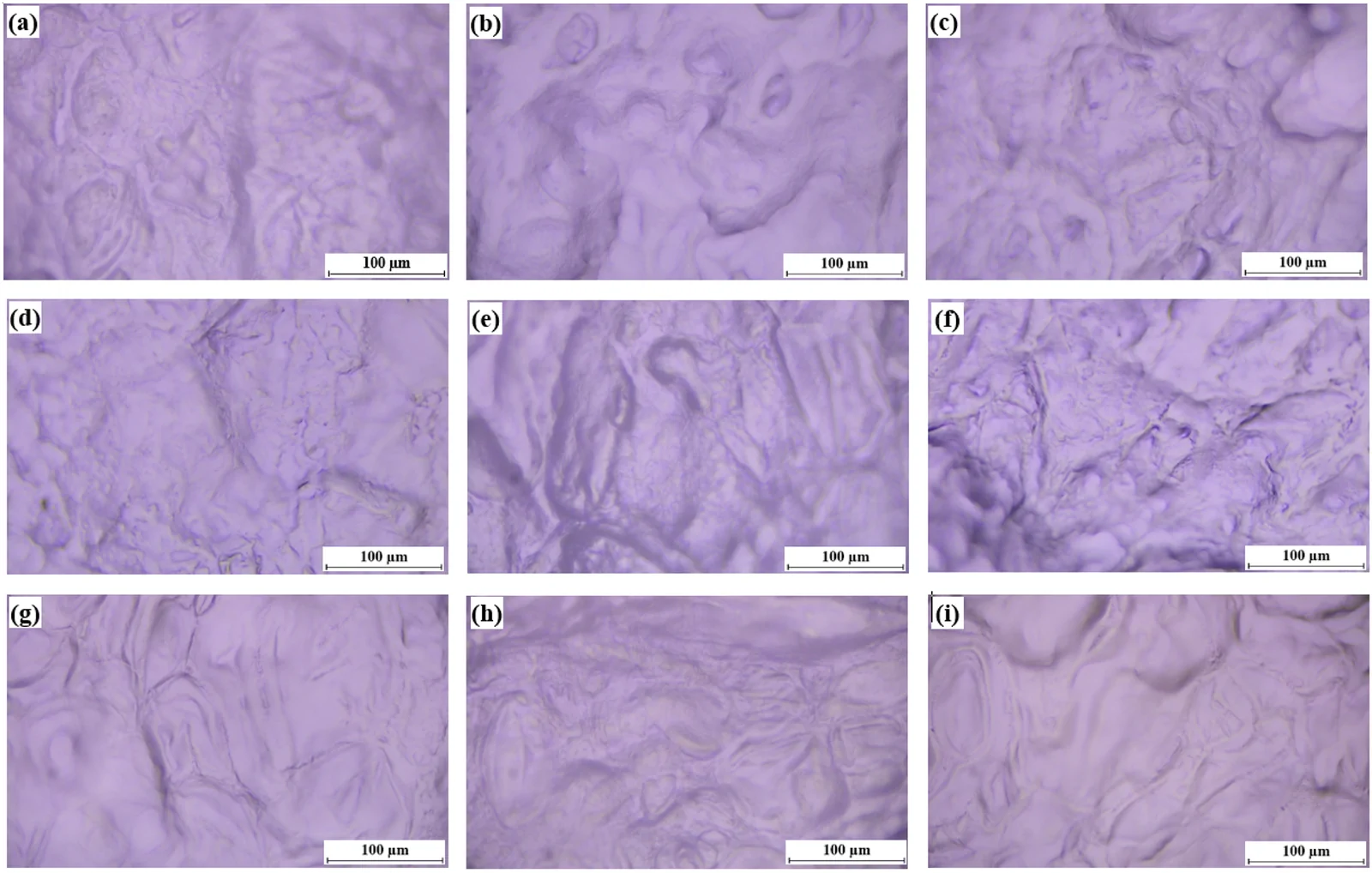
50× magnification micrographs of TPS films. (a) Control, (b) LGTPS, (c) CGTPS, (d) PGTPS, (e) PG1TPS, (f) PG2TPS, (g) GPTPS, (h) GP1TPS, and (i) GP2TPS.

**Figure 9:**
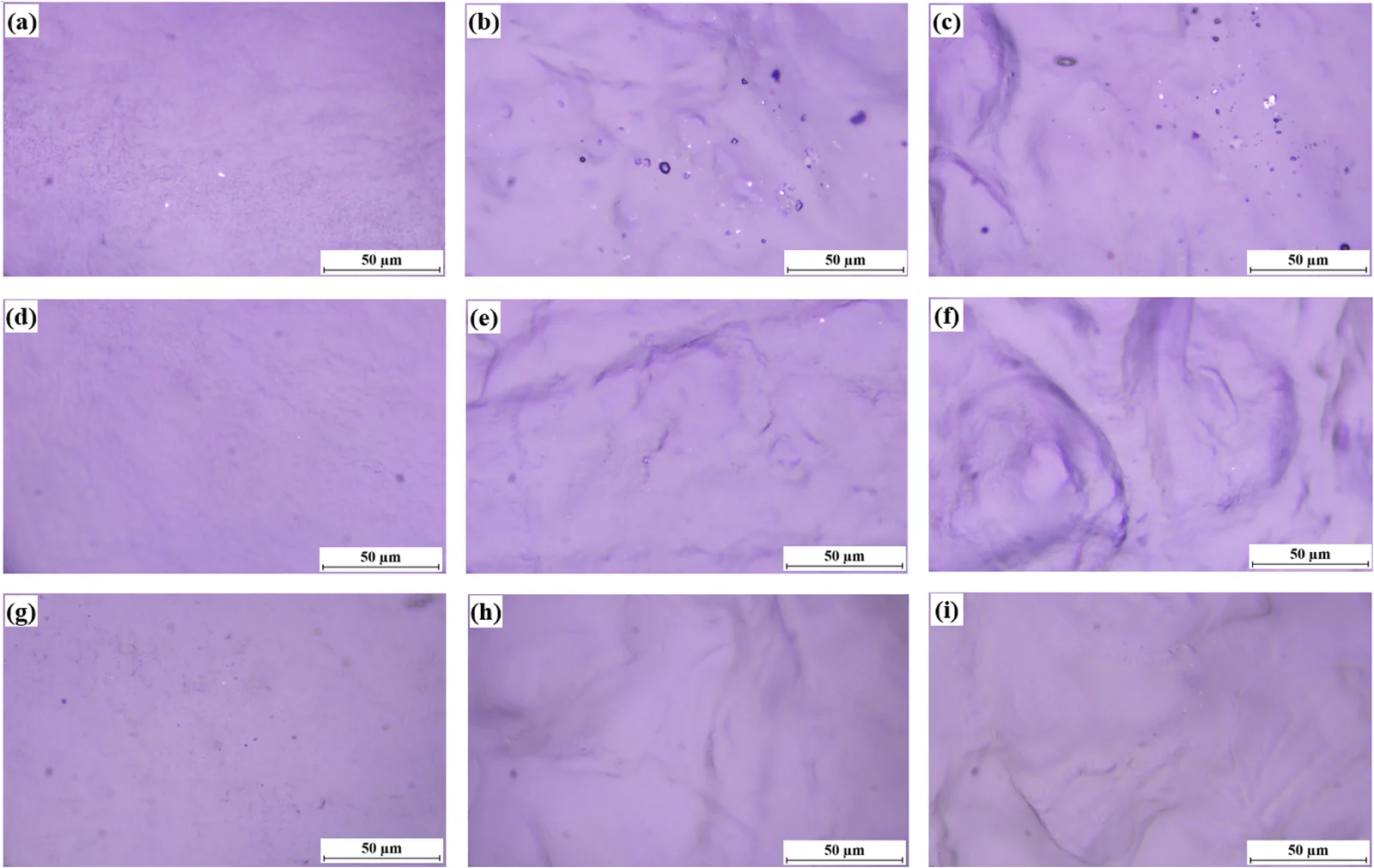
100× magnification micrographs of TPS films. (a) Control, (b) LGTPS, (c) CGTPS, (d) PGTPS, (e) PG1TPS, (f) PG2TPS, (g) GPTPS, (h) GP1TPS, and (i) GP2TPS.

### 3.10 FTIR Analysis of TPS Films

Figure 10a presents the FTIR spectra of TPS films plasticized with different glycerin types. All plasticized films display a broad absorption band in the 3000–3600 cm^–1^ region, corresponding to O–H stretching vibrations of hydroxyl groups in starch and glycerol.^27^ The breadth and intensity of this band indicate extensive inter- and intramolecular hydrogen bonding within the starch–glycerol network, which contributes to the structural integrity and functional properties of TPS films.^30^ Absorption bands near 2881 cm^–1^ correspond to aliphatic C–H stretching vibrations, primarily associated with CH_2_ groups present in the starch backbone and the glycerol-based plasticizers.^38^ New or markedly intensified absorption bands appear at approximately 1715 cm^–1^ and 1240 cm^–1^ in all plasticized films. The band near 1715 cm^–1^ is assigned to ester carbonyl (C = O) stretching, while the band around 1240 cm^–1^ corresponds to C–O–C asymmetric stretching within ester groups, which falls within the typical range for aliphatic and citrate esters (1300–1100 cm^–1^, particularly 1275–1185 cm^–1^).^20^ The presence of these bands suggests that citric acid underwent esterification with hydroxyl groups of starch, forming ester linkages within the TPS matrix. Such interactions can enhance molecular compatibility and improve structural cohesion in the plasticized starch network.^39^ At lower wavenumbers, the band at 994 cm^–1^, assigned to skeletal vibrations involving both C–O–C and C–C linkages within the polysaccharide backbone, shows increased intensity in the plasticized films. This change suggests modifications in starch–plasticizer interactions and short-range ordering within the starch matrix.^27^ A peak near 923 cm^–1^ is attributed to C–O asymmetric stretching of the α-(1 → 4) glycosidic linkage, indicating that the fundamental starch backbone structure is retained after plasticization.^38^ The band around 850 cm^–1^, associated with CH and CH_2_ deformation vibrations in starch, further supports the preservation of the core saccharide framework despite plasticizer incorporation and chemical modification.^38^ The H–O–H bending vibration at ∼1649 cm^–1^ shows reduced intensity in LGTPS, CGTPS, and GPTPS compared with the FTIR spectrum of the corresponding glycerin. Bands in the 1630–1600 cm^–1^ region are commonly assigned to the bending vibration of absorbed or coordinated water molecules.^20^ In starch-based films, a similar peak at 1642–1645 cm^–1^ has been attributed to hydroxyl groups associated with absorbed water, with possible contributions from O–H vibrations of glycerol plasticizer molecules.^27^ The attenuation of the 1649 cm^–1^ band in the plasticized TPS films therefore suggests a reduction in loosely bound water and possible reorganization of the hydrogen-bond network within the starch–plasticizer matrix. In PGTPS, however, this band is absent from the spectrum of pure PG (Figure 2). This is most likely associated with moisture uptake, as pharmaceutical-grade glycerol is highly hygroscopic and can contribute to the H–O–H bending signal in this region. Additionally, a distinct peak at 1558 cm^–1^, assigned to asymmetric COO^–^ stretching, is observed only in GPTPS, indicating retention of GP-derived carboxylate species within the TPS matrix. The lower intensity of this band compared with GP alone likely reflects dilution of these species within the starch–plasticizer network.

**Figure 10:**
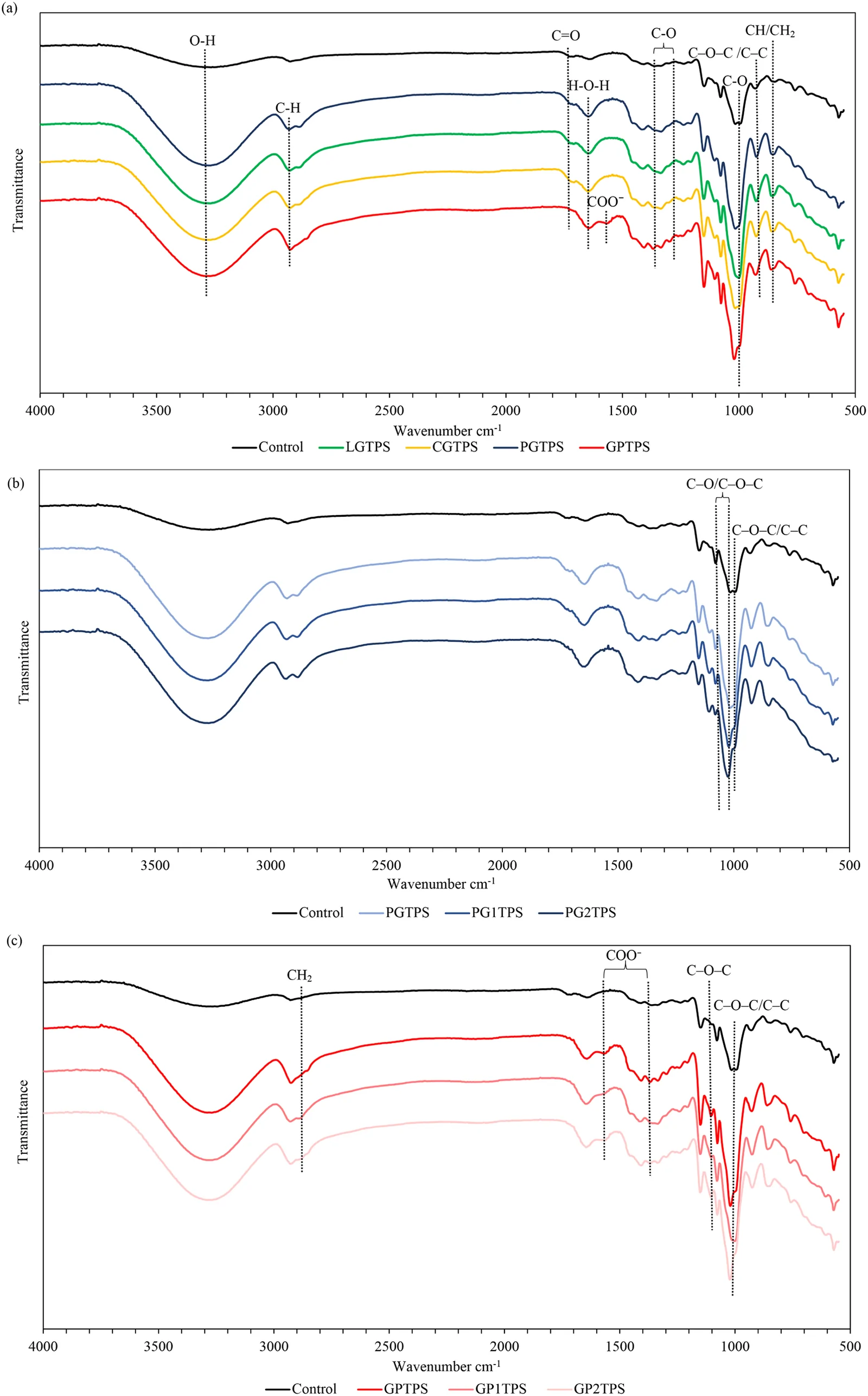
FTIR spectra of thermoplastic starch (TPS) films: (a) TPS films plasticized with different types of glycerin-based plasticizers; (b) PG-plasticized TPS films with different glycerin contents, and (c) GP-plasticized TPS films with different glycerin pitch contents.

The FTIR spectra of PG-plasticized TPS films show compositional changes with increasing glycerin content (Figure 10b). The bands at 1109 and 1070 cm^–1^ fall within the saccharide C–O/C–O–C stretching region, while the band at 994 cm^–1^ corresponds to skeletal C–C/C–O–C vibrations associated with the anhydroglucose ring. Similar assignments have been reported for starch-based films, where peaks in the 1030–990 cm^–1^ region are sensitive to the local environment of the anhydroglucose ring and the organization of the starch matrix.^38^ The progressive decrease in intensity of the 1109, 1070, and 994 cm^–1^ bands with increasing glycerin content suggests dilution of starch- and ester-related functionalities together with modification of the hydrogen-bond network. Such changes are consistent with the disruption of ordered starch domains and an increase in the amorphous character of the TPS matrix. These spectral variations therefore support the role of glycerin as a plasticizer, promoting greater molecular mobility within the starch–plasticizer network.

For GP-plasticized films, the CH_2_ symmetric stretching band at 2881 cm^–1^ increases in intensity from GPTPS to GP1TPS and reaches its maximum in GP2TPS, reflecting the increasing contribution of aliphatic CH_2_ groups associated with the added glycerin pitch (Figure 10c). In contrast, the COO^–^ bands at 1558 and 1370 cm^–1^ exhibit reduced intensity in GP1TPS, suggesting composition-dependent changes in the carboxylate environment and starch–GP interactions rather than a simple linear response. A similar nonmonotonic trend is observed for the C–O–C stretching band near 1100 cm^–1^, which is weakest in GP1TPS, while the 994 cm^–1^ skeletal band reaches its maximum at this formulation. This band corresponds to skeletal vibrations involving both C–C and C–O–C (glycosidic) linkages within the starch backbone. Together, these spectral variations suggest that the intermediate GP loading promotes a distinct microstructural reorganization of the starch–GP network, accompanied by changes in carboxylate speciation and short-range ordering. The sensitivity of the 1500–1600 cm^–1^ region (carboxylate vibrations) and the 800–1200 cm^–1^ fingerprint region (C–C/C–O stretching) to ionization state and local structural ordering has also been reported for citric-acid-modified thermoplastic starch films.^40^ This observation supports the interpretation that starch–GP interactions within the TPS matrix are strongly dependent on GP composition and loading.

Overall, the solvent resistance and degradation behavior of the TPS films were strongly influenced by the identity of the plasticizer. PG-plasticized films degraded most rapidly in both aqueous and organic media, whereas GP-plasticized films exhibited the highest resistance. This behavior reflects the influence of plasticizer composition, polarity, and molecular weight distribution on matrix stability. While FTIR analysis provided chemical evidence of plasticizer and water incorporation into the TPS network, the extent of plasticization in this study was primarily evaluated through macroscopic film behavior, including flexibility, tack, brittleness, solubility, thickness, and moisture retention. These parameters are widely recognized as practical indicators of plasticization in starch-based films. Direct thermal–mechanical measurements, such as glass transition temperature (Tg) determination or dynamic mechanical thermal analysis (DMTA), were not performed in the present study and represent a limitation of the work. Future studies will incorporate Tg and DMTA analyses to provide a more quantitative assessment of plasticization efficiency in GP-plasticized TPS systems.

### 3.11 Solvent Solubility Analysis

TPS films softened upon immersion in water as the starch matrix absorbed moisture but remained intact for removal, drying, and weighing without noticeable shrinkage. Figure 11a presents the weight loss of TPS films in water over five immersion intervals. Solubility in deionized water depended strongly on the glycerin plasticizer. The control (unplasticized) exhibited the lowest weight loss, reflecting the strong water resistance of a densely hydrogen-bonded, semicrystalline starch network.^41^ PGTPS and CGTPS showed higher solubility, indicating that purified and crude glycerin increase matrix hydrophilicity and chain mobility, thereby facilitating water penetration and dissolution. In contrast, GPTPS displayed the lowest solubility among the plasticized films, likely due to residual impurities and partial cross-linking in glycerin pitch, which limit water uptake. This observation is consistent with previous reports that increasing glycerol content enhances starch-film solubility by disrupting intermolecular hydrogen bonding.^27^ Overall, glycerin plasticization increased solubility, with the magnitude largely governed by the plasticizer’s polarity and composition.^31^ The observed variations in flexibility, brittleness, solubility, thickness, and moisture retention provide indirect evidence of plasticization in the TPS films, reflecting increased molecular mobility and reduced intermolecular hydrogen bonding within the starch matrix. Although direct thermal–mechanical measurements such as Tg or DMTA were not performed in the present study, the combined FTIR evidence and macroscopic film behavior provide consistent indications of effective plasticization in the TPS systems.

**Figure 11:**
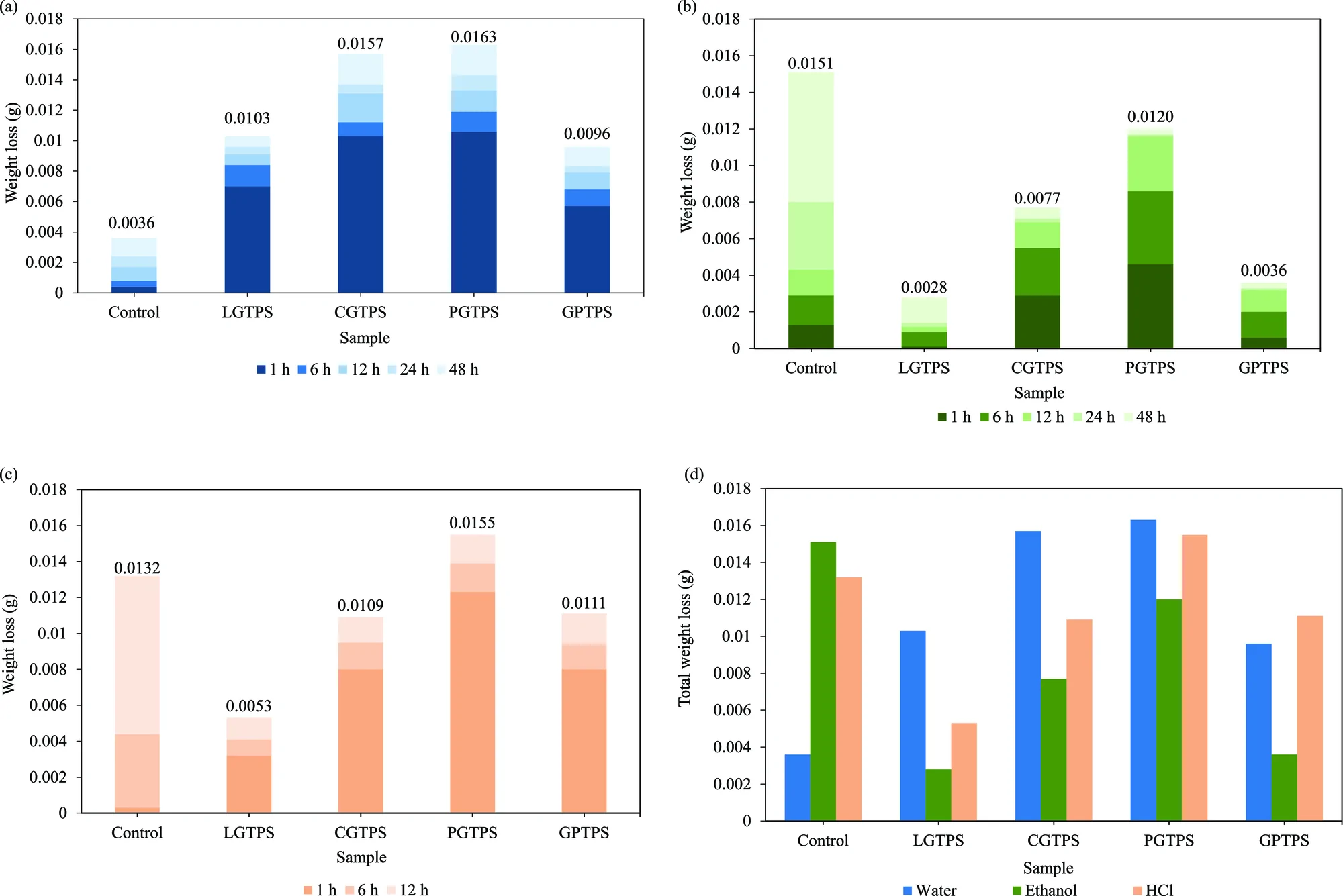
Weight loss of TPS films immersed in different solvents: (a) water and (b) ethanol over five intervals (1, 6, 12, 24, and 48 h); (c) HCl over three intervals (1, 6, and 12 h); and (d) total weight loss after immersion to the final measurable time point in each solvent. NaOH is not shown because all samples rapidly converted to soft gels within 1 h, preventing accurate gravimetric determination.

Ethanol immersion rendered the TPS films noticeably brittle, consistent with solvent-induced dehydration (loss of bound water) and partial extraction of plasticizer, which reduces film flexibility. The films remained intact and could be retrieved for drying and weighing without noticeable dimensional change. Ethanol solubility followed the order: control > PGTPS > CGTPS ≈ GPTPS > LGTPS (Figure 11b). The control film showed the highest mass loss because its unplasticized and dehydrated matrix fractured and dispersed readily. Among the plasticized films, PGTPS exhibited the lowest resistance, as glycerol’s high polarity and strong miscibility with ethanol promoted solvent uptake, disrupted starch–starch interactions, and facilitated plasticizer leaching.^27^ CGTPS and GPTPS displayed intermediate resistance, with GPTPS likely benefiting from higher-molecular-weight and less-polar components that enhance network cohesion. LGTPS showed the greatest resistance, which may be attributed to a more uniform starch–plasticizer interface and lower free volume that limits ethanol diffusion. Overall, the polarity, purity, and molecular-weight distribution of the plasticizer strongly influenced ethanol sorption and matrix stability. This observation is consistent with previous reports that glycerol-rich systems tend to exhibit greater hygroscopicity and solvent sensitivity than alternatives such as sorbitol or polyethylene glycol (PEG).^31^ From an application perspective, higher ethanol-induced mass loss (PGTPS and CGTPS) suggests faster degradation in alcohol-rich environments, whereas lower loss (GPTPS and LGTPS) indicates greater chemical stability.

Upon immersion in aqueous HCl, the TPS films softened, similar to the behavior observed in water, due to moisture uptake from the acidic medium. All films remained intact and could be retrieved for drying and weighing without noticeable dimensional change. However, after 24 h, the films had completely dissolved, producing a slightly white, cloudy dispersion consistent with solubilized or dispersed starch (Figure S2). Consequently, weight-loss measurements at 24 and 48 h were not determined. Acid solubility varied with plasticizer type (Figure 11c). PGTPS exhibited the highest total mass loss, followed by control, GPTPS, CGTPS, and LGTPS. The higher loss observed for PGTPS is consistent with the high polarity and purity of glycerol, which promotes stronger interactions with the acid medium, facilitates plasticizer migration, and increases chain mobility—mechanisms known to enhance the solubility of glycerol-plasticized starch films.^27^ Despite lacking a plasticizer, the control film also showed relatively high mass loss, likely because its tightly hydrogen-bonded and brittle matrix fragments and disperses more readily under acidic conditions. This behavior aligns with previous reports indicating that plasticizers reduce brittleness in starch films by weakening intermolecular hydrogen bonding.^31^ Among the plasticized samples, LGTPS showed the lowest mass loss, indicating the greatest resistance to acid attack. This behavior is consistent with a more homogeneous starch–plasticizer matrix that limits acid penetration. CGTPS and GPTPS displayed intermediate resistance, which may be attributed to the presence of higher-molecular-weight and less-polar fractions that reduce extractability and slow acid ingress by forming more cohesive and less permeable matrices. Overall, these results highlight that solvent resistance in starch films is strongly governed by plasticizer identity and formulation. Previous studies have similarly reported that lower hygroscopicity and higher molecular weight of the plasticizer phase can improve barrier properties and reduce permeation in thermoplastic starch systems.^31,41^

All samples partially dissolved and formed a very soft, jelly-like material after 1 h of immersion in NaOH (Figure S3). This transformation rendered the specimens unhandleable for drying or weighing. Attempts to recover residues by filtration were unsuccessful, even after extended drying at elevated temperatures. The gelled material remained highly water-laden, permeated the filter paper, and left no recoverable film (Figure S4). Consequently, gravimetric weight-loss measurements in 0.1 M NaOH could not be obtained. The rapid alkali-induced swelling and dispersion prevented reliable determination of solubility using the standard mass-loss method and therefore did not allow meaningful evaluation of the alkali resistance of the TPS films under these conditions. Alkaline media disrupt intermolecular hydrogen bonding within the starch matrix, promoting extensive swelling and dispersion; under sufficiently strong alkaline conditions, cleavage of α-1,4 and α-1,6 glycosidic linkages may also occur.^42^

Figure 11d summarizes the total weight loss of each TPS film after immersion in different solvents. In water, PGTPS exhibited the greatest mass loss, consistent with the high hydrophilicity of purified glycerin. In contrast, GPTPS showed the lowest solubility among the plasticized samples, indicating improved water resistance that may arise from the presence of higher-molecular-weight and less polar constituents in glycerin pitch.^31^

In ethanol and HCl, a similar trend was observed. PGTPS degraded most extensively, followed by CGTPS and LGTPS, whereas GPTPS again displayed moderate to high resistance. Notably, LGTPS showed the strongest resistance in ethanol, which may be associated with a more homogeneous starch–plasticizer matrix formed by high-purity glycerin.

Under alkaline conditions (NaOH), all films rapidly converted into soft, gel-like materials within 1 h, preventing gravimetric analysis and indicating the strong susceptibility of TPS to alkaline environments. Overall, chemical resistance was strongly influenced by plasticizer identity. PG-plasticized films degraded most rapidly, whereas GP-plasticized films exhibited the highest stability, suggesting that the latter may be more suitable for applications requiring improved moisture and solvent resistance.

Acidic and basic environments are known to alter the starch structure during processing and solvent exposure. Under acidic conditions, starch undergoes hydrolysis through the cleavage of glycosidic bonds, particularly in the amorphous regions of the granule, altering physicochemical properties such as solubility and pasting behavior.^43^ In contrast, alkaline treatment disrupts intermolecular hydrogen bonding and promotes extensive swelling by ionizing hydroxyl groups, thereby modifying the structural and functional behavior of starch.^44^ The dissolution and gelation behavior observed in acidic and alkaline media in this study are consistent with these established mechanisms.

Water should also be considered an intrinsic component of GP, and its role as a secondary plasticizer in starch systems is well recognized. Polyols such as water and glycerol disrupt intermolecular hydrogen bonding within the starch network, leading to structural relaxation and a reduction in the glass transition temperature of thermoplastic starch systems.^45^ Consequently, the differences in film behavior observed in this study are attributed to the combined effects of glycerol content, water, and impurities, rather than glycerol purity alone. The present work, therefore, evaluates glycerin pitch as a direct-use plasticizer rather than as a purified glycerol analogue.

## 4 Conclusion

GP contains a substantial inorganic fraction, as indicated by chloride-rich ash with elevated sodium and trace metals, while elemental analysis shows that its organic fraction is predominantly glycerol with a higher apparent oxygen content associated with moisture and oxygen-containing residues, distinguishing it from higher-purity glycerol grades. When used as a plasticizer in TPS films, GP produced materials with balanced plasticization, smooth surfaces, moderate elasticity, and the lowest solvent solubility among the tested systems, whereas films plasticized with pharmaceutical-grade glycerol exhibited overplasticization and surface defects at higher loadings. GP-based films also showed favorable physical integrity and visual appearance compared with films prepared using other glycerin grades. Overall, these results demonstrate that GP can function as an effective and low-cost plasticizer for TPS films, improving film stability and reducing solvent solubility while enabling the valorization of a biodiesel-derived industrial byproduct and supporting its potential use in starch-based biodegradable materials requiring enhanced durability and controlled solubility.

## Supplementary Material



## Data Availability

The data supporting the findings of this study are not publicly available due to their relevance to ongoing and unpublished work, but are available from the corresponding author upon reasonable request.

## Notes

Figure S1: Physical appearance of glycerine samples: (A) glycerine pitch (GP), (B) crude glycerine (CG), (C) laboratory-grade glycerine (LG), and (D) purified glycerine (PG); Figure S2: TPS films fully dissolved in 0.1 M HCl after 24 h; Figure S3: Physical appearance of TPS films after immersion in 0.1 M NaOH: (a) soft, jelly-like texture after 1 h; (b) dried state showing complete absorption into the filter paper, rendering the sample unpickable (PDF)
