# Supplementary material for: Characterization of Glycerin Pitch and Its Application as a Plasticizer in Thermoplastic Starch Films
Source: ACS Omega. 2026 Aug 8;11(33):48912–28. doi: 10.1021/acsomega.5c12175 (PMC13520560; doi:10.1021/acsomega.5c12175)
Supplement: Supplementary file 1 [file ao5c12175_si_001.pdf]

Characterisation of glycerine pitch and its application as a plasticiser in thermoplastic starch films

Shin-Yong Yeoh[1], Shee-Shyuan Lam[2], Shi-Yu Khor[3], Kun-Yi Andrew Lin[4], Fei-Yee Yeoh[2*]

[1]Food Technology Division, School of Industrial Technology, Building G07, Persiaran Sains, Universiti Sains Malaysia 11800 Pulau Pinang, Malaysia.

[2]School of Materials and Mineral Resources Engineering, Engineering Campus, Universiti Sains Malaysia, 14300 Nibong Tebal, Pulau Pinang, Malaysia.

[3]Tekno Impact PLT, 1, Jalan Setia Sentral, Bukit Mertajam, Pulau Pinang, Malaysia.

[4]Department of Environmental Engineering, National Chung Hsing University, 250 Kuo-Kuang Road, Taichung, Taiwan.

*Corresponding author: feiyee@usm.my

Supplementary files

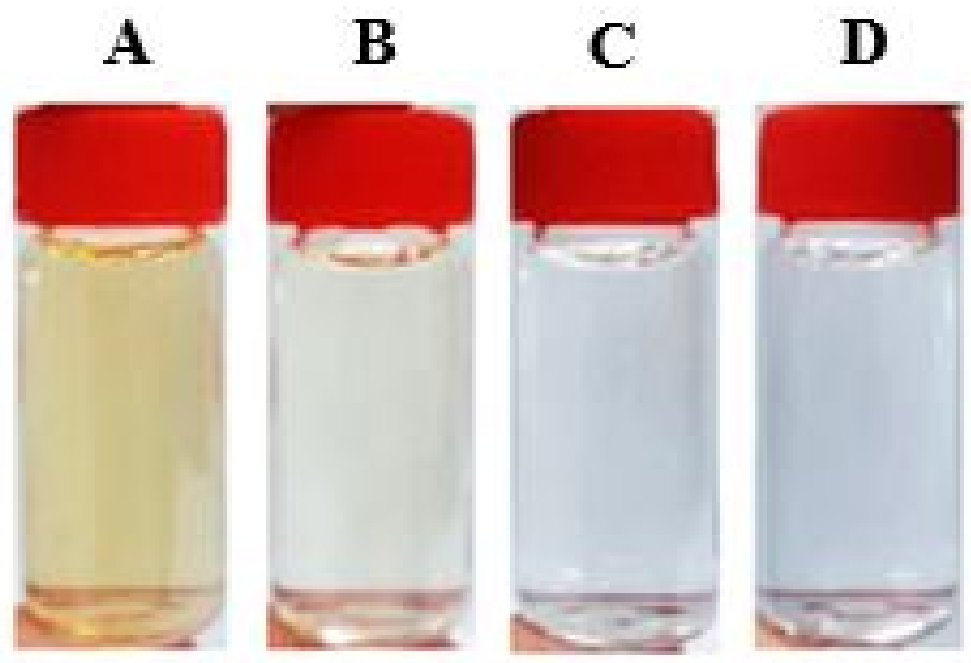

Figure S1. Physical appearance of glycerine samples: (A) glycerine pitch (GP), (B) crude glycerine (CG), (C) laboratory-grade glycerine (LG), and (D) purified glycerine (PG).

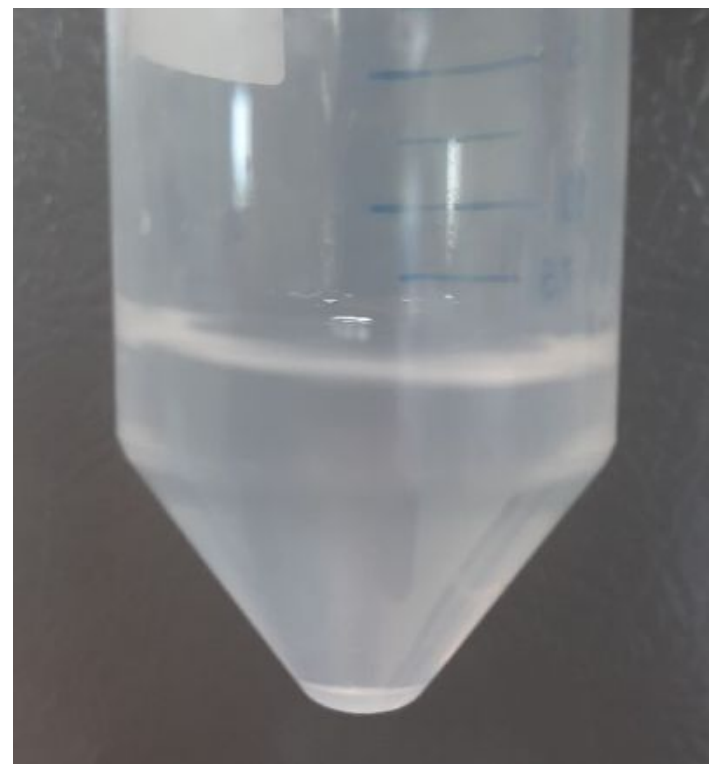

Figure S2. TPS films fully dissolved in 0.1 M HCl after 24 h.

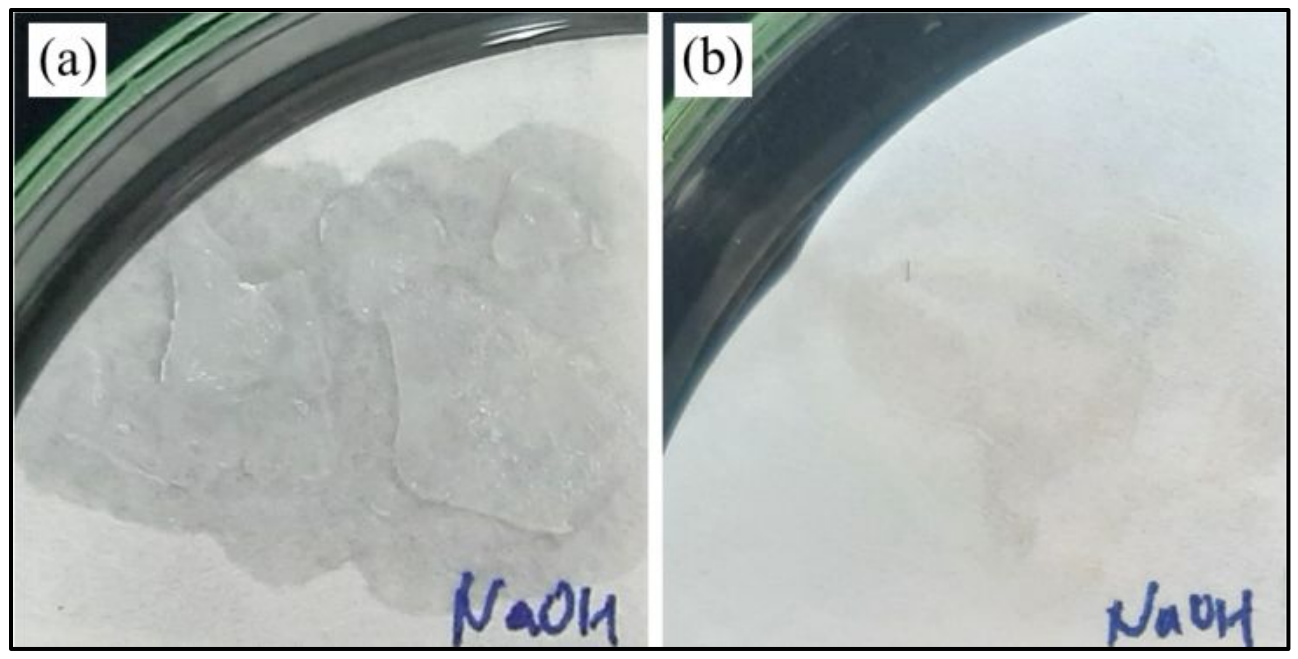

Figure S3. Physical appearance of TPS films after immersion in 0.1 M NaOH: (a) soft, jelly-like texture after 1 h; (b) dried state showing complete absorption into the filter paper, rendering the sample unpickable.
